# Dietary Knowledge, Attitude, Practices, and Perceived Dietary Barriers of Patients With Type 2 Diabetes: A Facility‐Based Cross‐Sectional Study, Khartoum, Sudan

**DOI:** 10.1002/hsr2.71591

**Published:** 2025-11-27

**Authors:** Rawan Rashid Mohamed, Amani Abdelrahman Sidahmed

**Affiliations:** ^1^ Faculty of Medicine University of Khartoum Sudan; ^2^ Department of Community Medicine University of Khartoum Sudan

**Keywords:** attitude, barriers, Diabetes mellitus, knowledge, practice, Sudan

## Abstract

**Background and Aims:**

In Sudan, the prevalence of diabetes was 18.9% in 2021. Diet is the cornerstone of diabetes management. Therefore, this study aimed to assess dietary knowledge, attitude, and practices (KAP) among patients with type 2 diabetes mellitus in Khartoum state, Sudan.

**Methods:**

A cross‐sectional study was conducted in the period from August 2022 to October 2022 among 365 diabetes patients recruited from four health centers in Khartoum state. A validated questionnaire was used. The collected data were analyzed using SPSS version 25. The chi‐square test and binary logistic regression analysis were used to identify variables that affect the dietary KAP of diabetes patients.

**Results:**

The majority of participants had good knowledge (99%), and a positive attitude (99.5%), but poor practice (80%). Female gender, coming from a primary health care center, and practice of physical activity were significantly associated with better practice scores. Cost and difficulty maintaining healthy dietary habits at social events were the major barriers to following dietary recommendations.

**Conclusion:**

The good knowledge and the positive attitude were not reflected in practice. This is contrary to the international dietary recommendations and reveals hindrances that prevent participants from applying what they know, and what they believe. Therefore, focusing on solving economic difficulties together with the other major barriers should be the attention focus of health care providers in order for the education programs to be effective.

**Abbreviations**
ADA: american diabetes association
DM: diabetes mellitus
IDF: international diabetes federation
KAP: knowledge, attitude, and practice
SP: sudanese pounds
USD: united states dollar
WHO: world health organization
(MNT): medical nutrition therapy

## 1 Introduction

Diabetes mellitus (DM) is defined as “a chronic disease caused by the reduction of insulin production by the pancreas or by the ineffective production of insulin” [1]. Low and high blood glucose levels can complicate it, with the latter leading to many long‐term complications such as cardiovascular diseases, renal failure, retinal diseases, neural diseases, and limb amputation [2]; which greatly affects patients' lives and productivity [3]. It is a worldwide problem affecting 537 million people in 2021 [4] and is estimated to affect 10.2% (578 million) by 2030% and 10.9% (700 million) by 2045 [5]. According to the World Health Organization (WHO), around 1 in 8 adults have diabetes worldwide [6], with around half of them being unaware of their disease.‬ In 2019, the number of diabetes patients was 19.4 million adults in Africa according to the International Diabetes Federation (IDF), and is expected to be 28 million by 2030 and 41.5 million by 2035. By 2030, it is expected that non‐communicable diseases will be at the top of the disease list, exceeding many common health problems, such as infectious diseases and nutritional diseases in the sub‐Sahara region [7]. The prevalence of diabetes in North Africa and the Middle East was 73 million in 2021 and is estimated to rise to 95 million by 2030 [4]. ‬‬‬‬‬‬‬‬‬‬‬‬‬‬‬‬‬‬‬‬‬‬‬‬‬‬‬‬‬‬‬‬‬‬‬‬‬‬‬‬‬‬‬‬‬‬‬‬‬‬‬‬‬‬‬‬

Sudan belongs to the North Africa and Middle East region, and according to the International Diabetes Federation (IDF), Sudan ranked among the top five countries for the highest number of adults living with diabetes in 2021, affecting 3.5 million people and a prevalence of 18.9%. The expenditure related to diabetes per person was 153.4 United States Dollars (USD) in Sudan in 2021 [4]. In Khartoum state, the prevalence of diabetes was estimated to be 11.6% in 2016, according to the Sudan STEPS Survey [8].

Nowadays, diabetes is becoming a major problem in both developing countries as for developed countries [9]. That increase in prevalence was attributed to lifestyle changes, including unhealthy dietary habits and sedentary lifestyles [10, 11]. Saturated fats, refined products, sugary drinks, and fast food, are all unhealthy food with increased consumption recently [12]. Some studies have reported an association between high sugar intake and the development of type 2 diabetes [7]. A follow‐up study of high‐risk patients demonstrated a lower rate of conversion to type 2 diabetes among those who practiced intensive lifestyle modifications [7]. The concept of Medical Nutrition Therapy (MNT) was first introduced by the American Diabetes Association (ADA) in 1994, meaning “applying special nutritional interventions for specific diseases in clinical practice” highlighting the importance of nutrition therapy besides drug therapy [7]. It takes into account the modification of diet to suit different cultures and religions, making it a science and an art [13, 14]. Patients with short disease duration can use MNT as the sole treatment [15]. Reduction in the need for medications and disease control are both benefits of dietary modifications [16], and this makes lifestyle modifications play a pivotal role in type 2 diabetes management [17].

The ADA emphasized the importance of dietary knowledge and its role in diabetes control if patients applied it [7]. Dietary knowledge is regarded as a key factor in changing unfavorable dietary practices, with a positive attitude towards dietary management being a vital determinant of the extent of commitment and degree of compliance to dietary recommendations [3, 15]. Healthy dietary practices contribute to the reduction of diabetes complications, enhance glycemic control of diabetes, and improve disease outcome [1, 16]. Poor dietary knowledge, attitude, and practice among patients with diabetes contribute to disease progression and the development of highly preventable complications [18, 19, 20]. In Sudan, the prevalence of diabetes showed a two‐fold rise between 2013 and 2021 [21], which was attributed to lifestyle changes among which dietary habits were the most important [22].

To our best understanding, the current state of dietary knowledge, attitude, and practice of patients with diabetes in Sudan is not completely known, as little has been published in this regard. Therefore, this study was conducted to assess the knowledge, attitude, and practices of patients with type 2 diabetes and identify their perceived barriers regarding dietary control of their disease in four diabetes centers in Khartoum state.

## 2 Methods

### 2.1 Study Area and Study Population

A descriptive, cross‐sectional, facility‐based study was conducted in the period from August 2022 to October 2022 in four centers in Khartoum state (Omer Ibn Al‐Khattab Health Center, Al‐Khatmia Health Center, Shambat Health Center, and Abdalla Khalil Diabetes Center). Those centers were chosen because they have the highest capacity for reviewing diabetes patients in Khartoum state according to Farag et al. [18]. Besides, there were no statistics from the Khartoum State Ministry of Health regarding health centers with the highest diabetes patient admission rate. The former three centers are primary health care centers, while the latter is a governmental diabetes center. The diversity in the level of the chosen centers is to discover whether the provision of dietary education is affected by the level of the health care center. Those centers are located in Khartoum, Omdurman, and Bahry cities, which are the three major cities that form the triangular capital of Sudan, Khartoum [23], making the sample representative to the target population. The study included patients with type 2 diabetes, aged 18 years or more, who had been diagnosed with diabetes for at least 1 month and accepted to participate in the study. Those who were severely ill, had communication difficulties, or had other types of diabetes like gestational diabetes and type 1 diabetes were excluded from the study.

### 2.2 Sample Size and Sampling Method

The total number of patients with type 2 diabetes attending those four centers was 1,300 per month, and the distribution of patients was 350, 450, 150, and 350 among Omer Ibn Al‐Khattab Health Center, Abdalla Khalil Diabetes Center, Al‐Khatmia Health Center, and Shambat Health Center respectively. A 20% nonresponse rate was added, giving a final sample size of 365 participants using the formula: *n* = N/1 + N (e^2^). Where *n* = sample size, *N* = target population = 1300, and *e* = margin of error (indicates the level of variability in responses i.e. permissible = 0.05 [24]). With the available data, Yamane's formula was the applicable one.

The number of participants from each center was calculated according to their proportion. Omer Ibn Al‐Khattab Health Center represented 25% of the total sample, Abdalla Khalil Diabetes Center represented 35%, Al‐Khatmia Health Center represented 15%, and Shambat Health Center represented 25%; therefore, the sample taken from each center was 91, 128, 55, and 91 participants respectively. At the level of each center, systematic random sampling was used.

### 2.3 Questionnaire Validation and Structure

A structured questionnaire, administered by face‐to‐face interview, was used. The questionnaire was administered to the patients in Arabic. It was designed by the authors based on the UK Diabetes and Diet Questionnaire (UKDDQ) [25], American Diabetes Association (ADA) dietary recommendations [7, 14], MH Mphasha et al. questionnaire [26], and another previous study [27]; and has been revised by a dietitian, three diabetes educators, and a statistician.

The questionnaire was pre‐tested on 10% (*n* = 30) of voluntary diabetes patients who were not included in the study to check the clarity, and length of the questionnaire, and to test reliability using the Cronbach alpha test which showed values of 0.734, 0.725, and 0.704 for the knowledge, attitude, and practice (KAP) questions respectively.

The questionnaire contained five sections: 11 items related to socio‐demographic and diabetes‐related data; 21 knowledge‐related questions with yes, no, and I don't know options; 6 attitude‐related questions with agree, neutral, and disagree options; 8 practice‐related questions with two options: 4–7 times a week, and 0–3 times a week, two questions were reversed coded to allow score calculation; and finally the perceived barriers section which was a multiple choice question.

Each correct answer (yes) in the knowledge questions was given 1, and the wrong answer (no or I don't know) was given 0. For the 21 knowledge related questions, the maximum possible score was 21, and the minimum possible score was 0. Likewise, for the 6 attitude related questions, each correct answer (agree) was given 1, and the wrong answer (neutral or disagree) was given 0. The maximum possible score was 6, and the minimum possible score was 0. For the 8 practice related questions, each correct answer (4–7 times a week) was given 1, and the wrong answer (0–3 times a week) was given 0. The maximum possible score was 8, and the minimum possible score was 0.

To calculate the knowledge score, the total score of each participant was calculated. Those who scored ≥ 50% of the highest possible knowledge score were considered to have a good knowledge, while those who scored < 50% of the highest possible knowledge score were considered to have poor knowledge according to previous studies [27, 28]. The percentage of good knowledge score = number of participants who had good knowledge score divided by the total number of participants and multiplied by 100. A similar method was used to calculate the percentage of poor knowledge score. Similar calculations were applied for the attitude and practices scores, with a positive attitude and a good practice corresponded to scoring ≥ 50% of the highest possible score, and a negative attitude and a poor practice corresponded to scoring < 50% of the highest possible score of each item.

### 2.4 Statistical Analysis

Data were entered into a Microsoft Excel 2019 spreadsheet and were checked for entry errors and coded to be ready for the analysis. Continuous variables, like age, were transformed into categorical variables in accordance with the BJUI guidelines (page 5) [29]. Descriptive statistics (frequencies and percentages) were used to describe socio‐demographic characteristics and presented in tables, while percentages were used to describe comorbidity status and perceived dietary barriers and presented in figures.

Statistical tests were performed with a level of significance of 0.05. The chi‐square test was used to determine the association between each of the KAP and socio‐demographic characteristics, testing whether dependent variables (level of dietary knowledge, attitude, and practices) can be predicted given the independent variables (type of health care facility, gender, age, educational level, occupation, marital status, income, duration of disease, having a family member with diabetes, practicing physical activity, receiving dietary education, and having comorbidity); and to test for association between the KAP elements. A *p*‐value > 0.05 (two‐sided) at a 95% confidence interval (CI) was considered to be statistically significant.

Binary logistic regression analysis was used to model the association and to predict the probability of the dependent variable controlling for the influence of multiple independent variables on the dependent variable, with quantification of association strength and direction given by the odds ratio, which was used as explanatory analysis. Adjusted odds ratio (AOR) at a 95% CI was considered to be statistically significant (two‐sided) with *p*‐value > 0.05. The data were analyzed using SPSS software for Windows (SPSS Inc. Chicago, IL version 25 was used for the analysis). The KAP elements are regarded as categorical variable hence the chi‐square test and logistic regression models were used as was done by M. Atuahene et al. (page 3) [30], and H. Adam et al. (page 3) [31].

The study was done according to the Helsinki Declaration. Ethical clearance was obtained from the Institutional Review Board of the Department of Community Medicine, Faculty of Medicine, University of Khartoum with the number COMMED 2022‐94‐45, according to the ethical guidelines at both national and institutional levels. A formal permission letter was obtained from the Khartoum State Ministry of Health and submitted to the Primary Health Center administration, and the Abdalla Khalil Diabetes Center administration. Verbal, informed consent was taken from each participant. Confidentiality and anonymity were maintained throughout the research.

## 3 Results

### 3.1 Socio‐Demographic Characteristics

**Figure 1:**
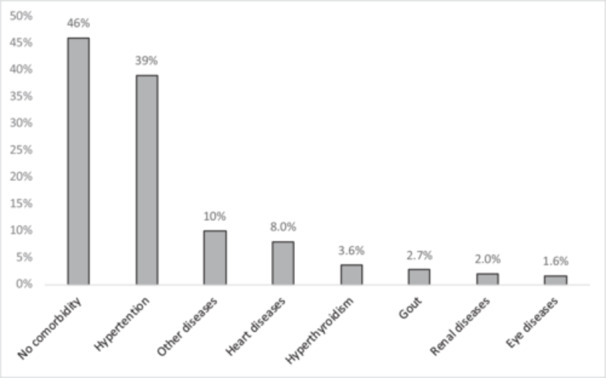
Comorbidities of patients with type 2 diabetes: A facility‐based study, Khartoum, Sudan (*n* = 365).

Out of 365 participants, 237 (65%) were recruited from primary health care centers, while 128 (35%) were from a tertiary diabetes center. Their ages ranged from 19 to 80 years, with a mean age of 56 (SD 13.4) years, and the majority of participants were females (57%, *n* = 209). A summary of socio‐demographic details is in Table 1. About half of the participants had comorbidities (54%, *n* = 197), with hypertension being the most frequent comorbidity (36%, *n* = 131) (Figure 1).

**Table 1:** Socio‐demographic characteristics of patients with type 2 diabetes: A facility‐based study, Khartoum, Sudan (*n* = 365).

Variables	Frequency	Percent
Health care facility		
Omer Ibn Al‐Khattab HC	99	27%
Al‐Khatmia HC	39	11%
Shambat HC	99	27%
THC center	128	35%
Gender		
Female	209	57.3%
Male	156	42.7%
Age		
< 40 years	49	13.4%
4010313860 years	171	46.8%
> 60 years	145	39.8%
Educational level		
Informal education†/illiterate	48	13%
Primary education	103	28%
Secondary education	131	36%
University or above	83	23%
Occupation		
un‐employed‡	249	68%
Employee	62	17%
Freelancer	55	15%
Marital status		
Single	35	9.6%
Married	272	74.5%
Divorced	15	4%
Widow	43	12%
Household monthly income		
< 50,000 Sudanese Pound (SP)§	219	60%
50,000–100,000 SP	115	31.5%
> 1000,000 SP	31	8.5%
Duration of disease		
< 5 years	90	24.7%
5–15 years	179	49%
> 15 years	96	26.3%
Do you have a family member with diabetes?		
Yes	288	79%
No	77	21%
Do you practice any form of physical activity?		
Yes	121	33%
No	244	67%
Did you receive any education regarding diet recommendations from health care providers?		
Yes	339	93%
No	26	7%
Do you have any comorbidity?		
Yes	196	54%
No	169	46%

Abbreviations: HC, health center; THC, tertiary health care (Abdalla Khalil Diabetes Center).

^†^ Informal education; any noncertified education (Khalwa and self‐learning (read and write).

^‡^ Un‐employed; including retired and housewives.

^§^ 1 USD = 569 Sudanese Pound.

### 3.2 Dietary Knowledge, Attitude, and Practice

Regarding knowledge of dietary recommendations, the majority (99%, *n* = 361) of participants had good knowledge, the vast majority had a positive attitude (99.5%, *n* = 363), while 80% (*n* =292) had a poor practice score. Almost all participants correctly answered the knowledge and attitude‐related questions. The knowledge, attitude, and practice sections are summarized in Tables 2, 3, 4 respectively. Of note, more than half (59%, *n* = 215) of participants did not consume sugary drinks regularly, and only 2.0% (*n* = 7) consumed fried food frequently. Breakfast is a frequently taken meal by almost all participants (99.7%, *n* = 364), and the majority of participants (83%, *n* = 303) admitted not following dietary recommendations frequently.

**Table 2:** Detailed dietary knowledge of patients with type 2 diabetes: A facility‐based study, Khartoum, Sudan (*n* = 365).

Dietary knowledge questions	Frequency	Percent
1. Banana, mango and orange contain high sugar		
Yes	336	92%
No/I don't know	29	8.0%
2. Strawberry, apple and watermelon contain low sugar		
Yes	349	96%
No/I don't know	16	4.0%
3. Diabetic patients can drink juices without sugar		
Yes	324	89%
No/I don't know	41	11%
4. It is better to take tea, hot drinks and coffee with sweetener than with sugar		
Yes	360	99%
No/I don't know	5	1.0%
5. Potato, pumpkin and corn are high in starch which ultimately change into sugar inside the body		
Yes	358	98%
No/I don't know	7	2.0%
6. Cucumbers, tomatoes, peppers, and carrots are good for diabetics		
Yes	364	100%
No/I don't know	1	0.0%
7. Rice and pasta are rich in starch that can raise blood sugar		
Yes	344	94%
No/I don't know	21	6.0%
8. If I want to eat bread I should choose whole grain bread		
Yes	298	82%
No/I don't know	67	18%
9. A diabetic patient can eat Kisra, Asida, and Qurrasa in small quantities (Sudanese food rich in starch)		
Yes	355	97%
No/I don't know	10	3.0%
10. Bean, peas, and, lentils are good for diabetics		
Yes	347	95%
No/I don't know	18	5.0%
11. Whole milk, yoghurt, cheese, and egg are rich in fats that can raise blood cholesterol levels		
Yes	348	95%
No/I don't know	17	5.0%
12. Nuts, oils such as sunflower and olive oil are examples of good fats		
Yes	358	98%
No/I don't know	7	2.0%
13. Pies, butter, margarine, cream and hydrogenated fats are bad fats		
Yes	347	95%
No/I don't know	18	5.0%
14. When preparing meat, it is recommended to remove visible fats from red meat		
Yes	360	99%
No/I don't know	1	1.0%
15. Fish provide a good source of protein, omega 3, and vitamin D		
Yes	363	99%
No/I don't know	2	1.0%
16. Eating chicken without skin is healthy		
Yes	359	98%
No/I don't know	6	2.0%
17. It is better to cook by grilling, baking, poaching or steaming instead of frying or roasting		
Yes	362	99%
No/I don't know	3	1.0%
18. Canned foods, restaurant foods should be reduced because of high salt content		
Yes	351	96%
No/I don't know	14	4.0%
19. Eating a large portion of food at once may lead to increased blood sugar		
Yes	355	97%
No/I don't know	10	3.0%
20. Diabetic patient is allowed to eat snacks		
Yes	362	99%
No/I don't know	3	1.0%
21. Dinner can prevent hypoglycemia		
Yes	342	94%
No/I don't know	23	6.0%

**Table 3:** Detailed dietary attitude of patients with type 2 diabetes: A facility‐based study, Khartoum, Sudan (*n* = 365).

Dietary Attitude questions	Frequency	Percent
1. Nutrition plays an important role in diabetes management		
Agree	365	100%
Neutral/Disagree	0	0.0%
2. Following dietary restrictions can delay or prevent complications of diabetes		
Agree	363	99%
Neutral/Disagree	2	1.0%
3. I will follow dietary restrictions if I have access to the recommended food		
Agree	360	99%
Neutral/Disagree	5	1.0%
4. When a person is labeled as diabetic, it is important for him/her to change his/her diet		
Important	210	58%
Neutral/No important	155	42%
5. How confident are you that you could change your diet to a healthy one?		
Confident	365	100%
Neutral/Not confident	0	0.0%
6. Are you concerned about your weight?		
Concerned	364	100%
Neutral/Not concerned	1	0.0%

**Table 4:** Detailed dietary practices of patients with type 2 diabetes: A facility‐based study, Khartoum, Sudan (*n* = 365).

Dietary practice questions	Frequency	Percent
1. How often did you eat vegetables?		
4–7 times a week	120	33%
0–3 times a week	245	67%
2. How often did you eat fruit?		
4–7 times a week	114	31%
0–3 times a week	251	69%
3. How often did you take sugary drinks? e.g. juices, soft drinks, sweetened tea, or coffee		
4–7 times a week	151	41%
0–3 times a week	214	59%
4. How often did you eat fried and fatty food? e.g. Whole‐fat dairy products, margarine, red meat, or chicken skin		
4–7 times a week	8	2%
0–3 times a week	357	98%
5. How often did you eat meat or chicken?		
2–4 times a week	141	39%
0–1 times a week	224	61%
6. How often did you eat breakfast?		
4–7 times a week	364	99.7%
0–3 times a week	1	0.3%
7. How often did you eat lunch and supper?		
4–7 times a week	4	1%
0–3 times a week	361	99%
8. How often did you follow health service providers' recommendations regarding diet?		
Always/most of the time	61	17%
Never or sometimes	304	83%

### 3.3 Factors Affecting Dietary Knowledge, Attitude, and Practice

The Chi‐square test showed no statistically significant association of knowledge or attitude with any of the socio‐demographic characteristics. However, it showed a significant association of practice with gender, age, educational level, level of healthcare center, and practice of physical activity. On further justification using binary logistic regression analysis; practice showed significant association only with gender, level of healthcare center, and practice of physical activity. Female patients had 2.26 (95% CI: [1.09–4.71], *p* = 0.002) times higher odds of having good dietary practice than male patients, those from a primary healthcare center had 2.34 (95% CI: [3.19–34.9], *p* = 0.0001) times higher odds of having good dietary practice compared to those from a tertiary healthcare center, and those who did not practice physical activity had 0.81 (95% CI: [0.21–0.97], *p* = 0.04) times lower odds of having good dietary practice than those who did. Further details are in Table 5.

**Table 5:** Binary logistic regression analysis of factors affecting dietary knowledge, attitude, and practices of patients with type 2 diabetes: A facility‐based study, Khartoum, Sudan (*n* = 365).

Variables	Knowledge		Attitude		Practice	
	OR (95% CI)	*p*‐value	OR (95% CI)	*p*‐value	OR (95% CI)	*p*‐value
Health care facility						
PHC center	2.31 (0.02–2.97)	0.28	1.56 (0.20–0.22)	0.21	2.34 (3.19–34.92)	0.0001*
THC center	1		1		1	
Gender						
Female	3.21 (0.06–0.08)	0.07	1.23 (0.21–0.23)	0.21	2.26 (1.09–4.71)	0.002*
Male	1		1		1	
Age						
< 40 years	1.73 (0.05–1.59)	0.15	2.80 (0.19–0.53)	0.35	1.23 (0.27–2.15)	0.13
40–60 years	2.53 (0.15–0.17)	0.16	3.15 (3.21–0.37)	0.12	2.16 (1.20–0.54)	0.46
> 60 years	1		1		1	
Educational level						
Informal education/illiterate	1.23 (0.32–126.39)	0.22	1.32 (1.12–0.32)	0.28	3.50 (1.59–1.81)	0.9
Primary education	1.32 (0.28–0.30)	0.29	2.13 (1.51–3.34)	0.31	2.81 (0.72–0.74)	0.73
Secondary education	3.12 (0.28–0.30)	0.3	1.32 (0.99–2.35)	0.43	2.41 (0.65–1.73)	0.38
University or above	1		1		1	
Occupation						
un‐employed	2.64 (0.08–10.37)	0.95	1.32 (0.564–0.439)	0.81	2.60 (0.215–0.237)	0.23
Employee	3.30 (0.99–1.00)	0.97	1.57 (0.04–2.69)	0.29	2.34 (0.71–2.68)	0.25
Freelancer	1		1		1	
Marital status						
Single	7.55 (0.13–16.37)	0.87	0.65 (0.60–0.71)	0.16	4.33 (2.96–1.27)	0.52
Married	9.82 (0.58–0.57)	0.9	0.75 (0.69–0.81)	0.17	4.23 (2.03–0.85)	0.51
Divorced	9.72 (0.57–0.56)	0.9	1.20 (214.5–175.6)	> 0.99	8.71 (2.51–2.12)	0.96
Widow	1		1		1	
Household monthly income						
< 50,000 Sudanese Pound (SP)	2.80 (0.012–3.624)	0.68	5.48 (1.723–3.246)	0.2	1.93 (0.248–1.226)	0.17
50,000–100,000 SP	3.50 (0.016–4.331)	0.69	5.34 (1.740–3.357)	0.22	6.78 (0.587–0.903)	0.18
> 1000,000 SP	1		1		1	
Duration of disease						
< 5 years	1.95 (0.02–1.67)	0.16	4.70 (0.46–0.48)	0.3	6.20 (0.35–0.20)	0.8
5–15 years	1.93 (0.25‐1.23)	0.17	8.37 (1.88–1.52)	0.38	3.80 (0.63–0.24)	0.82
> 15 years	1		1		1	
Do you have a family member with diabetes?						
Yes	4.87 (0.99–16.94)	0.92	1.63 (0.16–0.92)	0.55	4.35 (0.27–0.63)	0.47
No	1		1		1	
Do you practice any form of physical activity?						
Yes	1		1		1	
No	1.32 (0.08–16.02)	0.74	1.70 (0.92–0.93)	0.13	0.80 (0.21–0.97)	0.04*
Did you receive any education regarding diet recommendations from health care providers?						
Yes	5.05 (0.50–0.52)	0.4	1.15 (1.36–0.15)	0.13	1.29 (0.12–0.14)	0.35
No	1		1		1	
Do you have any comorbidities?						
Yes	9.23 (0.92–0.93)	0.53	7.35 (0.23–0.75)	0.28	2.03 (0.19–0.21)	0.47
No	1		1		1	

Abbreviations: CI, confidence interval; OR, odds ratio; PHC, primary health care; THC, tertiary health care.

* *p* < 0.05

### 3.4 Perceived Dietary Barriers

**Figure 2:**
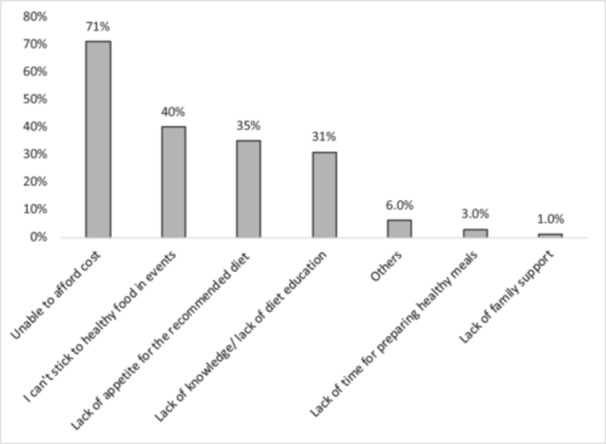
Perceived barriers towards dietary control of diabetes: A facility‐based study, Khartoum, Sudan (*n* = 365).

The high cost of healthy food was considered by most participants (71%, *n* = 259) as the most important barrier towards achieving diabetes control through diet followed by the difficulty to maintain healthy dietary habits when attending social events or going out with friends (40%, *n* = 146). Six percent of participants mentioned other barriers like priority of work over diet, carelessness, not thinking that diet can help control diabetes, and difficulty remembering dietary recommendations. Further details in Figure 2.

## 4 Discussion

This study found that the level of dietary knowledge was good with most participants (99%) having a good knowledge score. This is similar to the findings of a Malaysian study with 87% of participants having good knowledge scores on lifestyle modifications including diet [32]. On the contrary, an Ethiopian study found poor knowledge scores in 52% of participants. The difference in results can be explained by the finding that the majority of participants in that study (83%) considered lack of knowledge/lack of diet education as a major barrier [27]. A Sudanese study [31], found sufficient knowledge about diabetes self‐care activities, and another Sudanese study found good knowledge in 55% of participants [18]. The higher score in our study might be attributed to the increased availability of diet‐related information on different sources like media as stated in different studies [27, 33, 34].

This study found no statistically significant association of knowledge with any of the socio‐demographic characteristics. Similarly, a study in Pakistan [35] found no association with different characteristics like age, gender, occupation, educational level, and family history. On the other hand, several studies found knowledge to be significantly associated with different factors like educational level, gender, marital status, and household monthly income [31, 35]. Differences in results can be attributed to the very high knowledge score in this study, making participants with different socio‐cultural characteristics score high regardless of their socio‐cultural status.

As far as attitudes are concerned, the vast majority of participants (99.5%) had positive attitudes. Similarly in Malaysia, 98% had a positive attitude towards lifestyle modifications including diet [32]. On the other hand, 62% of participants in China had a negative attitude [36], and only 41% of participants in Pakistan had a positive attitude [35]. The difference in results can be attributed to the difference in content and number of used questions, and the difference in type of answer provided. Regarding the condition in Sudan, a study found that 87% of participants admitted that diet has a great effect on diabetes control which reflects a positive dietary attitude [18]. Another study found that 79% of participants had a positive attitude [31]. This 20% difference in results from our study can be attributed to the smaller sample size of 238 in the latter study.

This study found no statistically significant association of attitude with any of the socio‐demographic characteristics. This was the same result of Adam et al. in Sudan [31]. The finding of no association can be explained by what Adam et al. said: “Health education messages provided to the patients by the health care providers perhaps play an important role in developing positive attitudes towards the recommended diets irrespective of their socio‐demographic background” [31].

Regarding dietary practice, this study found it to be poor in 80% of participants. This finding is similar to a study conducted in Saudi Arabia which found a poor practice score with 87% of participants not following any special diet [37]. Another study at Services Hospital Lahore, Pakistan found that 82% of participants had poor dietary practice scores [35]. On the other hand, dietary practice scores were good in more than half of the participants (58%) in Jabber Abulizz Hospital in Khartoum, Sudan [31]. The difference in results can be attributed to the difference in sample size (238 participants), study area (only one center in that study), and used tool (that study asked only about whether participants followed the recommended diet or not which may not reflect the actual practice status of participants). Farag et al. found that 67% of participants in Sudan had sufficient practice regarding diet [18]. The difference in results can be attributed to the difference in study objectives with that study focusing on diabetes self‐care activities including diet, while our study focused only on diet.

Dietary practice in this study was significantly associated with gender, level of healthcare facility, and practice of physical activity. Females were 2.26 (95% CI: 1.09–4.71) times more likely to have good practice scores compared to males. This is similar to the findings in China [36], and Brazil [38], but unlike the results in Pakistan [35], nor Sudan [31]. The difference can be explained by the finding that in recent years, females tend to be more careful than males regarding their diet and weight [39, 40]. This study found a significant association between the level of the healthcare facility and practice scores with participants from a primary healthcare center having 2.35 (95% CI: 3.19–34.9) times higher odds of having good practice scores compared to those from a tertiary healthcare center. Farag et al. also found a difference in results with the difference in the level of healthcare facility [18]. This might be attributed to the finding that the financial burden is more on patients from tertiary health centers [41, 42], which is a major barrier to good dietary practice as is found in this study. This study also found that patients who did not practice physical activity were 0.81 (95% CI: 0.21–0.97) times more likely to have bad practice scores. Not many studies examined the relationship between dietary practice and the practice of physical activity to compare it with the findings of this study.

This study showed no statistical association between knowledge, attitude, and practice. Similarly, Farag et al. found no association between knowledge and practice [18], similar to the findings of a study in Thai [15]. The finding of no association in our study means that the very high knowledge and attitude scores were not translated into good practice which reflects that there were barriers that need to be considered. On the contrary, Adam et al. found an association between practice and both knowledge and attitude in Sudan [31]. The difference might be attributed to the burden of economic challenges in Sudan that increased over time as demonstrated by several studies [43, 44, 45]. Another study found an association between knowledge and practice [46]. The difference in results might be due to the difference in the study population with that study conducted among adolescents.

When it comes to the perceived dietary barriers, the high cost of the recommended diet was considered by most participants (71%) as the major barrier, which is similar to another Ethiopian study which found that more than half (57%) of participants considered the cost of healthy food as the main barrier [27]. A study in Malawi also documented cost as a major barrier for most participants [47]. This reflects the economic difficulties faced by participants of those countries as mentioned by a study in Sudan [43], and another study stated that the cost of a diabetes diet is much more than that for non‐diabetes [48]. Thus understanding patients' views is equivalent in importance to the provision of dietary education programs as stated by Jalilian et al. [49], and it addresses the need for policies and programs that support low‐resource countries. Next to the economic barrier was the difficulty sticking to a healthy diet at social events, family gatherings, and when going out with friends with 40% of participants admitting this difficulty. This obstacle accompanied Sudanese patients even outside their motherland as shown by a study conducted on Sudanese patients residing in Australia [50]. Similarly, most participants in Singapore perceived eating out as a major barrier [51]. Mphwanthe et al. found that many participants faced difficulties deciding what to eat during work and travel [47], and in South Africa, participants experienced the same difficulty in sticking to a healthy diet in gatherings for fear of disclosure [26]. Therefore, care providers need to exert more effort to enable diabetes patients to continue their diet while maintaining their social communication. Lack of appetite for the recommended diet was a major barrier for 35% of the participants of this study and those in Singapore [51]. Matpady P et al. observed that some participants experienced cravings for sweets, especially after their diagnosis [52]. Such a problem can be mitigated by educating patients when it is allowed to consume a few sweets. About one‐third (31%) of participants considered lack of dietary knowledge as a barrier which is similar to the results of a Malawian study [47], while in Ethiopia, 12% considered lack of knowledge as a major barrier [27]. Though this contradicts the results of this study (a good knowledge score of 99%), it can reflect dissatisfaction with provided education programs or lack of knowledge regarding how much to eat, but not what to eat as mentioned by P. Roth et al. [47]. Hence, it is important to have feedback from patients to clarify how they perceive education programs and to find ways to check the effectiveness of such programs.

## 5 Conclusions

This study found a good knowledge score of 99%, and a positive attitude score of 99.5%, while a poor practice score of 80%. This gap reflects that good knowledge and positive attitudes were not sufficient to be reflected as good practice. This is alarming that primary healthcare centers and diabetes centers provide continuous diabetes education in the absence of reflection in practice. Female gender, coming from a primary health care center, and practice of physical activity were associated with better dietary practice scores. Economic challenges and bad diet‐related social habits were perceived by most participants as major barriers, thus understanding patients' perspectives is a key factor in the success of education programs.

### 5.1 Strengths and Limitations

This study provided an introductory approach to studying the barriers to the application of dietary knowledge among diabetes patients, with the sampling technique makes the results generalizable to the larger population. As it was a facility‐based study, this will not cover the portion of the population that does not visit health centers, highlighting the need for a population‐based study. The close‐ended questions used in the questionnaire provide some guidance to participants compared to open‐ended questions.

## Recommendations

Diabetes education programs should provide more attention on how to enable patients with diabetes to overcome bad dietary habits at social events. More attention should be attracted to addressing the economic barrier, and not only focusing on providing diabetes education without checking patients' practice. There is a need for a qualitative study to throw light on the actual obstacles that hinder dietary practice.

## Author Contributions

**Rawan Rashid Mohamed:** determined the study design, and study area, designed the questionnaire, conducted the study, analyzed data, and wrote the manuscript. **Amani Abdelrahman Sidahmed:** supervised the whole process including conceptualizing the research questions, designing the study, and editing/reviewing the manuscript.

## Conflicts of Interest

The authors declare no conflicts of interest.

## Transparency Statement

The lead author Rawan Rashid Mohamed affirms that this manuscript is an honest, accurate, and transparent account of the study being reported; that no important aspects of the study have been omitted; and that any discrepancies from the study as planned (and, if relevant, registered) have been explained.

## Data Availability

The authors confirm that the data supporting the findings of this study are available within the article [and/or] its supplementary materials.
